# Multilayer perceptron neural network-genetic algorithm for modeling *Nicotiana tabacum* leaf quality

**DOI:** 10.1371/journal.pone.0330370

**Published:** 2025-10-07

**Authors:** Mohammad Reza Najafi, Mohammad Ali Aghajani, Naser Safaie, Siamak Rahmanpour

**Affiliations:** 1 Department of Plant Protection, College of Agriculture Sciences and Food Industries, Science and Research Branch, Islamic Azad University, Tehran, Iran; 2 Plant Protection Research Department, Golestan Agricultural and Natural Resources Research Center, AREEO, Gorgan, Iran; 3 Department of Plant Pathology, Faculty of Agriculture, Tarbiat Modares University, Tehran, Iran; 4 Department of Oil Seed Research, Seedling and Seed Research Institute, Karaj, Iran; Nuclear Science and Technology Research Institute, IRAN, ISLAMIC REPUBLIC OF

## Abstract

The global industry of tobacco (*Nicotiana tabacum* L.) is a profitable one comprising various products, including cigars, cigarettes, chewing tobacco, and smokeless tobacco. The internal quality of the cigarettes is highly related to the chemical components of tobacco leaves and shreds. Blue mold severity (BMS), chlorophyll (Chl), total nitrogen (N), sugar (S), nicotine (Nt), chloride (Cl), and potassium (K) contents of tobacco leaves are linked to the flavor and taste of cigarette products. A precise analysis of the effects of these factors would open the door for improving farmer income in low- and middle-income countries. In this study, BMS, Chl, N, S, Nt, Cl, K, green weight (GW), dry weight (DW), and leaf quality of four cultivars, including Bergerac, Bell, Burly, and Basma, were evaluated during two growing seasons. Bell displayed the highest leaf quality in two growing seasons. Multiple linear regression, stepwise regression, ordinary least squares regression, partial least squares regression, principal component regression, and multilayer perceptron neural network-genetic algorithm (MLPNN-GA) were used for the prediction of tobacco leaf quality responding to BMS, Chl, N, S, Nt, Cl, K. MLPNN-GA models displayed higher prediction accuracy compared with the best regression model according to R^2^ for MLPNN-GA vs. regression models were: Bergerac; 1.00 vs. 0.82, Bell = 1.00 vs. 0.41, Burly = 1.00 vs. 0.68, Basma = 0.94 vs. 0.68, and all cultivars = 0.94 vs. 0.66. The close match between the predicted and actual data validated the superior efficiency of the developed MLNNP-GA models for predicting tobacco leaf quality responding to BMS, Chl, N, S, Nt, Cl, K. Analysis of the developed MLPNN-GA models showed that Bergerac, Bell, Burly, and Basma leaf quality was most sensitive to BMS. MLPNN-GA was demonstrated to be a practical mathematical tool for predicting tobacco leaf quality in response to its chemical components and BMS.

## Introduction

Tobacco (*Nicotiana tabacum* L.), belonging to *Solanaceae* family, is a key economic crop and model plant for scientific research [1]. It can thrive in a variety of soil and climatic conditions [2]. The global tobacco industry is a profitable one comprising various products, including cigars, cigarettes, chewing tobacco, and smokeless tobacco [3]. Bergerac, Bell, Burly, and Basma are four Tobacco cultivars. Bergerac genotype originates from the Bergerac Tobacco Institute in southwestern France. The breeding programs there initially focused on resistance to Tobacco Mosaic Virus (TMV) and later adapted to combat blue mold outbreaks in the 1960s [4]. Bell genotypes (especially Bel-W3) were developed in the United States and are widely used as bioindicators of ozone (O₃) sensitivity. Bel-W3, in particular, has been used globally since 1962 to detect phytotoxic levels of ozone due to its high sensitivity. These lines are standard in environmental and physiological studies [5]. Burley tobacco originated from a natural mutation in White Burley tobacco in the United States. This mutation led to a plant with lighter, air-cured leaves that had a lower sugar content compared to other tobacco types. Burley became widely cultivated due to its unique curing characteristics and flavor profile, making it popular in cigarette blends [6]. Basma tobacco is a dark air-cured tobacco traditionally grown in the Eastern Mediterranean region, especially Turkey and Greece. It belongs to the Oriental tobacco group and is characterized by small leaves with a rich aroma. Basma genotype evolved through long-term selection under specific climatic and soil conditions, resulting in its distinct genetic and phenotypic traits [7].

The quality assessment of flue-cured tobacco, as a cash crop, is a sensory one of flavor style and quality consisting of agglomeration, gas diffusivity, aroma quality and quantity, softness, sweetness, dryness, miscellaneous gas, irritation, and aftertaste [8–11]. A key disadvantage related to these sensory factors determining tobacco quality are highly dependent on the person’s experience and senses.

The cigar tobacco quality is closely tied to the leaf-inherent chemical components. Sugar (S), chloride (Cl), nicotine (Nt), potassium (K), and total nitrogen (N) contents of raw materials are linked to the flavor and taste of cigarette products [12]. Nitrogen (N) and potassium (K) play crucial roles in determining the quality of tobacco leaves. Adequate nitrogen promotes vegetative growth, increases leaf area, and enhances chlorophyll and protein content, leading to higher yields. However, excessive nitrogen can delay leaf maturity, reduce sugar content, and negatively affect leaf texture and combustibility [13–15]. Potassium, on the other hand, significantly improves leaf burn quality, color, and aroma by enhancing sugar accumulation and balancing nitrogen uptake. A deficiency in potassium results in poor leaf texture, reduced burning characteristics, and increased susceptibility to diseases. Therefore, maintaining a balanced N:K ratio is essential for optimizing both the yield and quality of tobacco leaves [13–15]. Also, chlorophyll (Chl) concentration affects the quality of cigar tobacco. Chlorophyll content in the leaves is a key metric of plant health and is essential for the biosynthesis of aroma precursors [16]. The degradation of chlorophyll can affect the color of cigar leaves and the quality of cigar tobacco. Blue mold caused by *Peronospora tabacina* is the most widespread fungal disease of tobacco and the main limiting factor of tobacco cultivation [17,18]. In addition to the qualitative loss (yield and yield components), quantitative characteristics and chemical compounds of tobacco, including total alkaloid content, sugar, and nicotine, are significantly affected by this fungal disease [19,20]. Resistance or susceptibility to blue mold (*P. tabacina*) significantly influences the chemical composition, physical appearance, and overall quality of tobacco leaves. Resistant cultivars often produce healthier leaves with minimal disease damage. However, resistance mechanisms may alter metabolic pathways, leading to increased levels of defense-related compounds such as duvatrienediols (DVTs) and T-phylloplanins, which can negatively affect the flavor, aroma, and burning properties of the cured leaves [21,22]. Susceptible cultivars, if uninfected, may exhibit higher sensory and chemical quality, but are at greater risk of severe leaf damage, necrosis, and poor grading during disease outbreaks, drastically lowering market value [23]. Overall, tobacco price is determined by the factors that influence its leaf quality. The quality of tobacco leaves is influenced by various factors and their interactions, making classification a challenging task. A precise analysis of the effects of the factors would open the door for improving farmer income in low- and middle-income countries, but assessing it is complex and costly. Mathematical models and digital image processing offer automated grading methods, facilitating the evaluation and prediction of tobacco quality. Multivariate statistical techniques, namely multiple linear regression (MLR), stepwise regression (SR), principal component regression (PCR), partial least squares regression (PLSR), and ordinary least squares regression [OLSR; [24–26]] were used to explore the relationship between tobacco quality and its leaf chemical composition and blue mold severity (BMS). Traditional modeling techniques, including regression models, display insignificant nonlinear fitting ability [25–27]. Artificial intelligence (AI) handles challenges that cannot be addressed using traditional modeling techniques. Artificial intelligence describes computer programs qualified to do complex tasks that were previously only possible for human intelligence. Artificial neural networks (ANNs) are potent tools in the field of AI, which mimic human intelligence to think in a simplified way to process information and shed light on complex situations involving ambiguity and uncertainty [28–30]. ANNs acquire intelligence by uncovering hidden patterns and relationships through experience [29]. Multilayer perceptron neural network (MLPNN) is a powerful tool for resolving complex nonlinear issues. It can handle large data sets, make predictions fast after training, and achieve the same level of accuracy even with sparse data [31]. However, there are a lot of issues with ANN design and training. A few hidden neurons lead to low accuracy rates, whereas an abundance of hidden neurons lengthens training times and causes data overfitting [32]. Furthermore, another major issue that has a direct impact on the model’s performance is the MLPNN structure’s weight allocation. Weights are directly affected by the learning algorithm parameters, including learning rates, hidden node and layer number, memory tap number, and also network topology. Hybrid models, which couple the ANN pattern recognition abilities and the exploratory search of the optimization techniques, including genetic algorithm (GA), can address these complex issues [GA; 27, 33]. GA is a well-known search algorithm that makes excellent solutions to problems and has been applied to bioprocess optimization [26,27,34,35]. GAs use bio-inspired operators, including selection, crossover, and mutation, to make superb optimization solutions [36]. GA starts by creating a random population of potential search solutions called chromosomes. The algorithm selects the superior search solutions (the fitter chromosomes) to be included in the next generation through a roulette wheel selection technique. The selected search solutions/chromosomes experience a crossover operation and create new search solutions [offspring chromosomes, 37]. The search solutions obtained by GA improve over time; GA needs no auxiliary or derivative information, is a superior parallel algorithm, can optimize discrete and continuous functions, and multi-objective problems, and can search through a large search space [38]. MLPNN-GA addresses several limitations commonly associated with traditional regression methods [39,40]: Non-Linearity: Traditional regression often assumes a linear relationship between variables, but MLPNN can more effectively model complex, nonlinear relationships. Feature Interactions: Regression methods may struggle to capture interactions between features unless these interactions are explicitly included. MLPNN can naturally learn these interactions through its layered architecture. High Dimensionality: As the number of features increases, traditional regression techniques can become less effective. Nevertheless, MLPNN-GA can manage high-dimensional data better by learning from the data structure rather than relying on a preset form. Overfitting and Generalization: Traditional regression is prone to overfitting, especially with small sample sizes or noisy data. However, MLPNN-GA employs genetic algorithms for optimization and regularization techniques that help improve the generalization of unseen data. Robustness to Outliers: Traditional regression methods can be sensitive to outliers, which can skew results; nonetheless, the training process in MLPNN can be more robust to such anomalies through appropriate design and training strategies. Model Selection and Hyperparameter Optimization: MLPNN-GA uses genetic algorithms to optimize the neural network’s architecture (such as the number of layers and neurons) and the hyperparameters (like learning rate), thereby automating the model selection process. The integration of the Multilayer Perceptron Neural Network (MLP) with the Genetic Algorithm (GA) provides a powerful approach for predictive modeling, especially in complex and nonlinear domains, such as chemical composition-based price estimation. This hybrid model offers several key advantages [41–44]: 1. Optimal Parameter Tuning: GA efficiently optimizes the weights, biases, and hyperparameters of the MLP, overcoming limitations of conventional training methods that often suffer from local minima and slow convergence. 2. Enhanced Predictive Performance: By combining GA’s global search capability with MLP’s nonlinear mapping power, the model achieves superior accuracy and generalization compared to standalone MLPs or other machine learning algorithms. 3. Robustness in Modeling Nonlinear Relationships: The hybrid model effectively captures complex and nonlinear interactions within the data, which is critical for accurately linking chemical constituents to market prices. 4. Reduced Dependence on Manual Hyperparameter Selection: GA automates the tuning process, minimizing the need for extensive trial-and-error and expert intervention during model development. 5. Adaptability and Scalability: The MLP-GA framework is flexible and can be tailored to various datasets and problem scales, making it suitable for diverse agricultural product pricing challenges. This research was conducted to (a) to develop regression and MLPNN-GA models for predicting leaf quality of tobacco (*N. tabacum*) according to input variables “S, Cl, Nt, P, N, Chl, and BMS”, (b) to evaluate the performances of the MLPNN-GA and regression models regarding prediction accuracy of tobacco leaf quality (output variable), and (c) find the most critical input variables determining tobacco leaf quality.

## Materials and methods

### Experimental design

Field experiments were performed in the main tobacco-growing regions of Tirtash, in the north of Iran, during two growing seasons, i.e., 2015 and 2016. Tobacco fields are located at 36° 45’ N, 53° 44’ E at an altitude of 14 m. Average rainfall and temperature were 46.50 mm and 17.7 ^◦^C for 2015 and 71.11 mm and 18.0 ^◦^C for 2016 growing seasons, respectively. Each experiment was planned based on a randomized complete block design with 50 replicates. The experiments were conducted using four cultivars including Bergerac C, Bell 61−10, Burly 21, and Basma 178−2 (S1 Fig in S1 File). Each plot for Bergerac, Bell, and Burly included two 12.0 m long rows (0.5 m between plants) spaced 1.0 m apart. Basma plots consisted of two rows spaced 1.0 m apart with 0.3 m between plants in rows (25 plants in each 7.2 m long row).

Blue mold severity (BMS) was recorded according to the scale described by Cooperation Centre for Scientific Research Relative to Tobacco [CORESTA; 45]. Leaf nitrogen (N) content was determined according to the Kjeldahl method [46]. Also, leaf chlorophyll (Chl) content was measured by a chlorophyll meter. Besides, reducing sugar (S) content of leaves was determined using a titration of ferricyanide [47]. Furthermore, leaf nicotine (Nt) content was determined as described previously [48]. Additionally, leaf chloride (Cl) content was determined using Mohr’s Method [49]. Moreover, leaf potassium (K) was extracted through the calcination route [50]. Tobacco leaves dried at 105 °C to constant weight to provide the dry weight.

It is noteworthy that the price of tobacco leaves (per kg DW) was used as a measure of leaf quality. The price of tobacco leaves (per kg DW) was transformed to a z-score to avoid the effect of the scale caused by the currency of different countries. The mean and standard deviation of the tobacco leaf price dataset were 1579996.5 Iranian Rials kg^-1^ DW and 452320.3, respectively.

Combined analysis of variance (ANOVA) and separate ANOVA were used to study BMS, Chl, N, S, Nt, Cl, K, GW, DW, and the quality of four tobacco cultivars in two growing seasons. ANOVA and Mean comparisons using least significant difference (LSD) were conducted by SAS (SAS 9.1, 2003) and the graphs were made by GraphPad Prism 9 (GraphPad Prism 9, 2020) software.

### Model development

The datasets were normalized using Box-Cox transformation [51] before running the machine learning system. Principal component analysis (PCA) detected no outliers. The performance of each tested model on the dataset (100 and 400 data lines for each cultivar and all ones, respectively) was calculated using a five-fold cross-validation method with 10 repeats, and the model with the highest prediction accuracy for unknown data from the dataset was determined.

### Multilayer perceptron neural network (MLPNN) model

MLPNN modeling was used to define the effects of blue mold severity (BMS), sugar (S), chlorine (Cl), nicotine (Nt), potassium (P), total nitrogen (N), and chlorophyll contents on the quality of *N. tabacum* leaves.

MLPNN, one of the most widely used deep learning architectures, is made up of three layers: input, hidden, and output layers [52,53]. The input layer, which contains the input neurons, redirect input data to the next layer. The hidden layer is the key computation layer in MLPNN, which maps the complex relationships in data using mathematical functions. The output layer computes predictions using the outputs received from the last hidden layer. In MLPNN, all neurons in one layer connect to each neuron in the next layer using weighted connections. Also, bias (*β*), a parameter of hidden and output neurons, is a threshold that allows the neuron to adjust its activation level.

**Fig 1:**
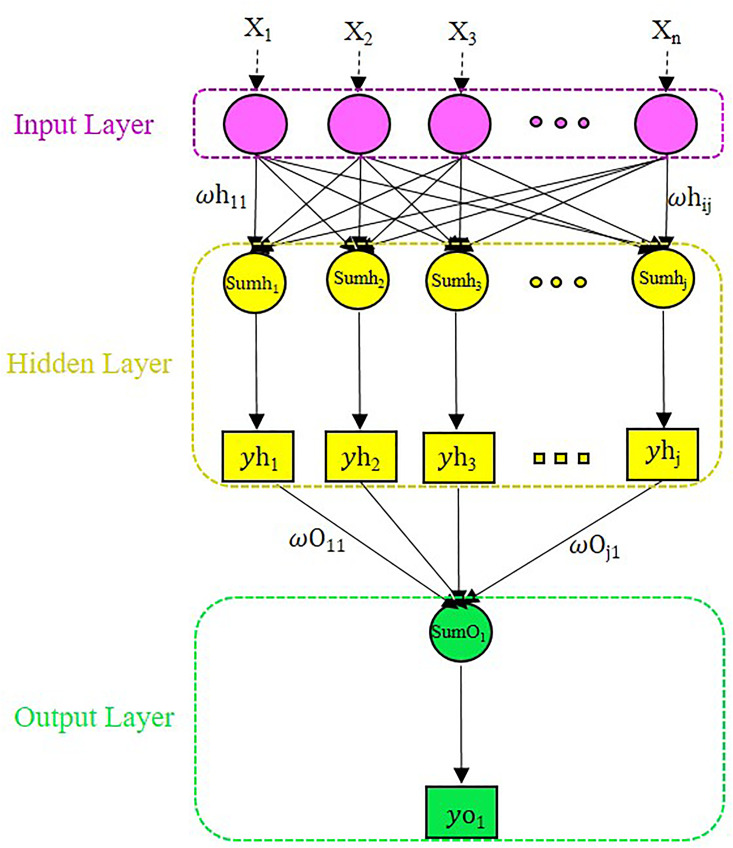
Schematic of multilayer perceptron neural network (MLPNN) architecture.


Fig 1 shows the topology of an MLPNN with *n* input neurons, one hidden layer containing *m* neurons, and one output neuron. ωh_ij_ signifies the weights connecting the input neurons to hidden ones. ωo_*j*1_ are the weights connecting the hidden neurons to output neurons. *β*_k_ denotes biases. The hidden layer performs two operational functions, i.e., summation and transfer (activation) ones. The summation function given in Eq. (1) is used to compute an output “Sum_j_” using a hidden neuron j.

$${Sumh}_{j}=\sum_{i=0}^{n}{(\omega h}_{ij}\;\times \;X_{i})+\;\beta_{j}\;$$ (1)

where ωh_ij_ signifies the weight connecting an input neuron *i* to the hidden neuron *j*, *X*_i_ denotes the output of the input neuron *i* feeding hidden neuron *j*, and *βj* define the bias of hidden neuron *j*.

The activation function maps the summation function result using mathematical functions. The most-used activation function is the sigmoid function calculated by Eq. (2).


$${yh}_{j}=f\;\left({Sumh}_{j}\right)=\frac{2}{1+\;e^{-2{Sumh}_{j}}}-1$$ (2)

where yh_j_ is the final output of the hidden neuron *j*. This output feed the output layer.

Also, output layers uses summation and activation functions. Summation function for output layer containing a single output neuron is computed by Eq. (3).


$${Sumo}_{1}=\sum_{j=0}^{m}{(\omega o}_{j1}\;\times \;{yh}_{j})+\;\beta_{m+1}\;$$ (3)

Where yh_*j*_ denotes the output of a hidden neuron *j*, and ωo_*j*1_ signifies the weight connecting the hidden neuron *j* to the output neuron. *β*_m+1_ is the output neuron bias.

Finally, the result predicted by MLPNN is determined using the activation function (Eq. 4).


$${yo}_{1}=f\;\left({Sumo}_{1}\right)=\frac{2}{1+\;e^{-2{Sumo}_{1}}}-1\;$$ (4)

### Genetic algorithm

**Fig 2:**
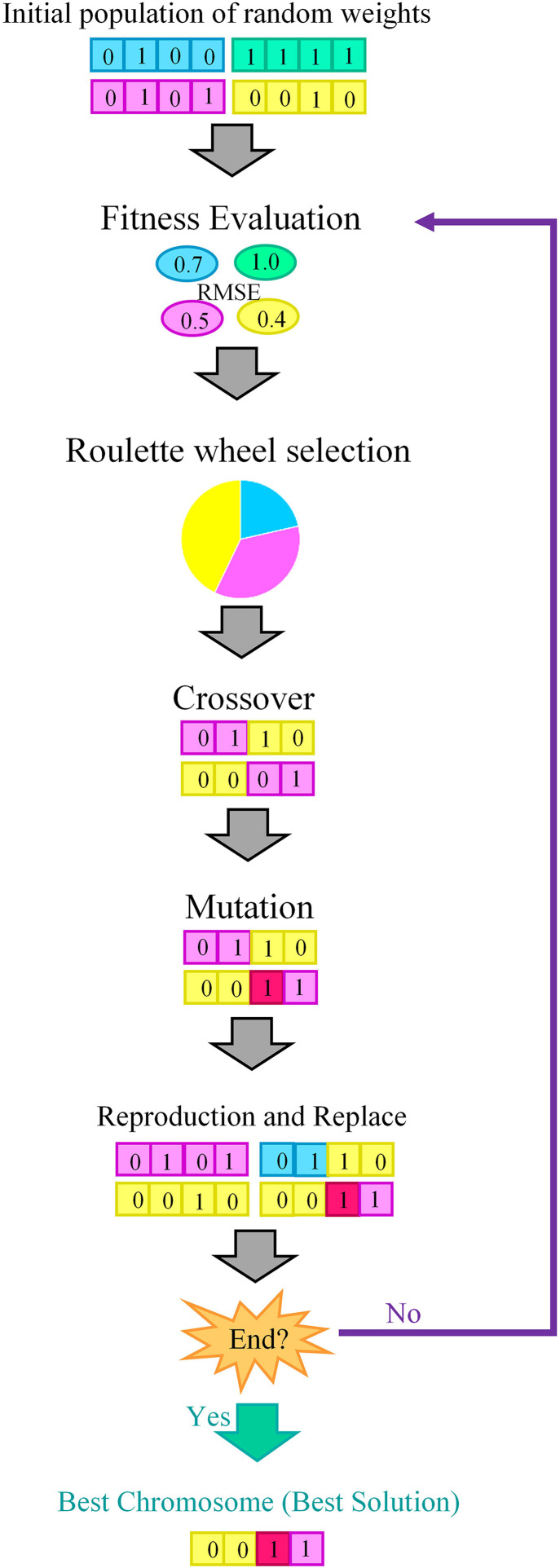
Schematic representation of genetic algorithm (GA) as an evolutionary optimization algorithm.

MLPNN architecture, including the biases and weight of hidden and output layers, was optimized using GA. An initial population of solutions (called individuals), usually created at random, is the starting point for the genetic algorithm [54]. The standard operators of GA are: (1) Initialization: An initial population (P) of chromosomes (solutions) is randomly selected, (2) Fitness evaluation: The performance of each chromosome in the initial population was evaluated by the fitness function, (3) Selection: a new population was selected from the initial population using roulette wheel selection method [55], (4) Crossover: the new individuals (new solutions) was created from the selected fittest chromosomes using crossover, (5) Mutation: the new individuals (new solutions) was generated using randomly modifying the one allele of chromosomes, (6) Replace: new offspring was placed in the new population, and (7) Test: if the end condition is met, stop and return the best solution in the current population. Otherwise, repeat steps 2–7 to identify the population with the highest fitness (Fig 2).

Root mean square error (RMSE) is a quadratic function and strongly penalizes when the MLPNN output is far from expected [56]. Therefore, RMSE was used as a fitness function to optimize MLPNN architecture.

An initial population, crossover rate, mutation rate, and generation number were set to 50, 0.85, 0.01, and 500, respectively, [27] to establish the fittest MLPNN structure.

**Fig 3:**
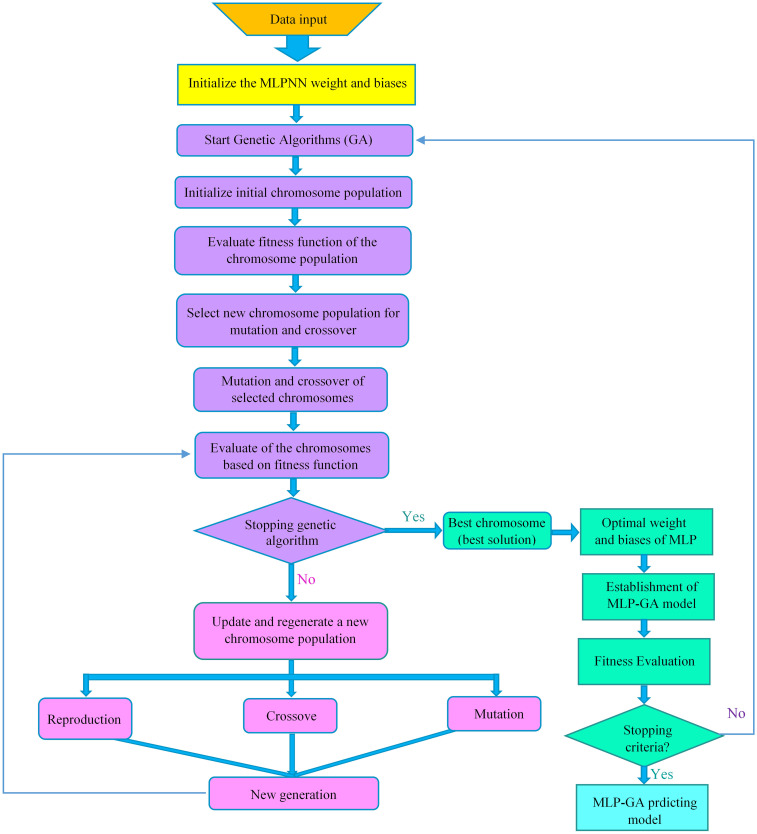
Flowchart of integrating multilayer perceptron neural network (MLPNN) with genetic algorithm (GA) to optimize MLPNN architecture.

Fig 3 illustrates the optimization strategy used to establish the optimal MLPNN architecture.

Statistical criteria of coefficient of determination (R^2^; Eq. (5)), root mean square error (RMSE; Eq. (6)), and mean absolute percentage error (MAPE; Eq. (7)) were used to assess the performance of MLPNN-GA hybrid model.


$$R^{2}=1-\frac{\sum_{i=1}^{n}{\;\left(y_{est}-\;y_{act}\right)}^{2}}{\sum_{i=1}^{n}{\;\left(y_{act}-\overset{―}{y}\right)}^{2}}\;$$ (5)

$$RMSE=\;\sqrt{(\sum_{i=1}^{n}{\left(y_{est}-y_{act}\right)}^{2})/n}\;$$ (6)

$$MAPE=\;\frac{1}{n\;}\sum_{i=1}^{n}\left|\frac{\left(y_{act}-y_{est}\right)}{\left(y_{act}\right)}\right|\times 100\;$$ (7)

where “n” is the number of data, “y_act_” signifies the actual values, and “y_est_” denotes the predicted values.

### Sensitivity analysis

Sensitivity analysis was conducted on MLPNN-GA models to find out the degree of the importance of the model input variables (BMS, Chl, N, S, Nt, Cl, and K) on the model output variable (leaf quality). Variable sensitivity error (VSE) was used to assess the sensitivity of a model to a specific input variable by measuring the model performance (using RMSE) when that particular variable is removed or unavailable, essentially showing how much the model’s accuracy suffers without that input data. Variable sensitivity ratio (VSR) value was determined by dividing VSE by MLPNN-GA model error (RMSE value) when all of the input variables were at hand. The estimated VSR values were then rescaled so that they fell within a range of 0–1. The highest importance variable of the model was the one with the highest VSR [26,27,30,57].

MATLAB (R2010a; MATLAB, 2010) and XLSTAT (XLSTAT, 2017) were used to develop and assess the MLPNN-GA and regression models, respectively, and GraphPad Prism 9 (2020) was used to create the visualizations.

## Results

### Effects of two growing seasons and cultivars on blue mold severity, chlorophyll, nitrogen, sugar, nicotine, chloride, potassium, green weight, dry weight, and quality of *Nicotiana tabacum* leaves

The effects of cultivars (Bergerac, Bell, Burly, and Basma) and interaction ones of growing seasons × cultivars on all the studied characteristics (BMS, Chl, N, S, Nt, Cl, K, GW, DW, and quality) were significant (S1 Table in S1 File). Two growing seasons (2015 and 2016) significantly affected BMS, Chl, Nt, K, GW, DW, and quality (S1 Table in S1 File). The significant interaction effects of growing seasons and cultivars indicated that the cultivars affected BMS, N, Chl, S, Nt, Cl, K, GW, DW, and quality differently depending on growing seasons, and vice versa (i.e., growing seasons affected BMS, Chl, Nt, K, GW, DW, and quality differently at each cultivar). Because of this significant interaction, the effects of cultivars were analyzed on each growing season.

**Fig 4:**
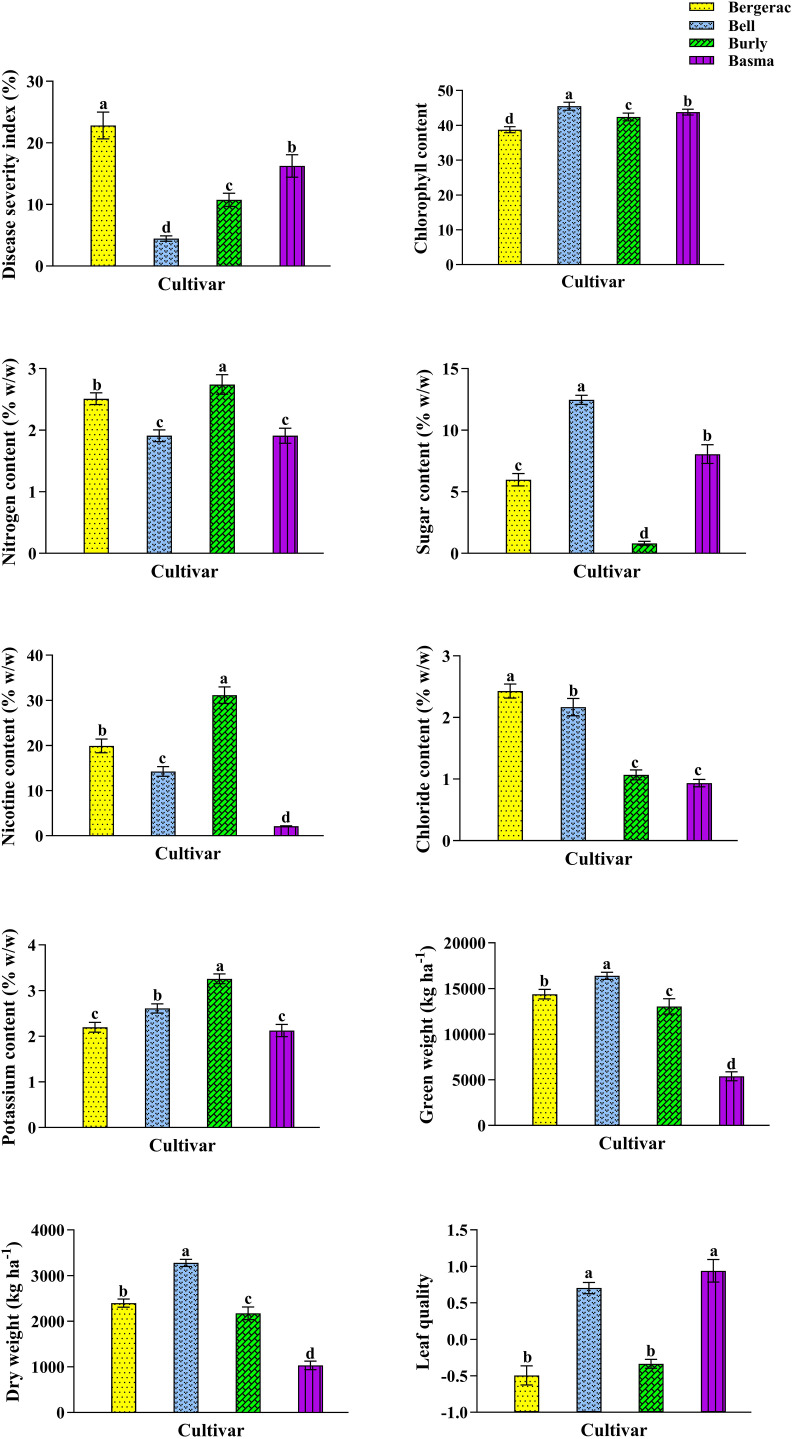
The characteristics of *Nicotiana tabacum* cultivars, including Bergerac, Bell, Burly, and Basma, in 2015 growing season. Mean values are given, standard error are represented by vertical lines. Means followed by the same letter are not significantly different (*p ≤ 0.05*).

Four cultivars in the 2015 (S2 Table in S1 File) and 2016 (S3 Table in S1 File) growing seasons significantly affected all the studied characteristics. The highest BMS in 2015 was recorded in Bergerac leaves with an average of 22.82% (Fig 4). Bell showed the lowest BMS (4.46%) in 2015, i.e., 80.46% lower than that in Bergerac (Fig 4). The highest Chl in 2015 (45.50) was measured in Bell leaves, i.e., 17.45% higher than that in Bergerac leaves showing the lowest Chl (38.74; Fig 4). Also, Burly leaves had the highest N, Nt, and K with an average of 2.74, 31.13, and 3.26% (w/w), respectively (Fig 4). Besides, the highest S (12.46% w/w), GW (16390.54W w/w), and DW (3278.11% w/w) in 2015 were recorded for Bell leaves, which were 14.23, 2.04, and 2.17 times recorded for Burly, Basma, and Basma, respectively (Fig 4). It is noteworthy that Burly, Basma, and Basma displayed the lowest S, GW, and DW, respectively. Additionally, the highest Cl content belonged to Bergerac leaves (Fig 4). Bell and Basma had the highest quality with an average of 0.8213, i.e., 40.19% higher than that in Bergerac and Burly leaves (Fig 4). It is critical to note that 40.19% is based on the main scale of measurement in our data set with the mean and standard deviation of 1579996.5 Iranian Rials kg^-1^ DW and 452320.3, respectively.

**Fig 5:**
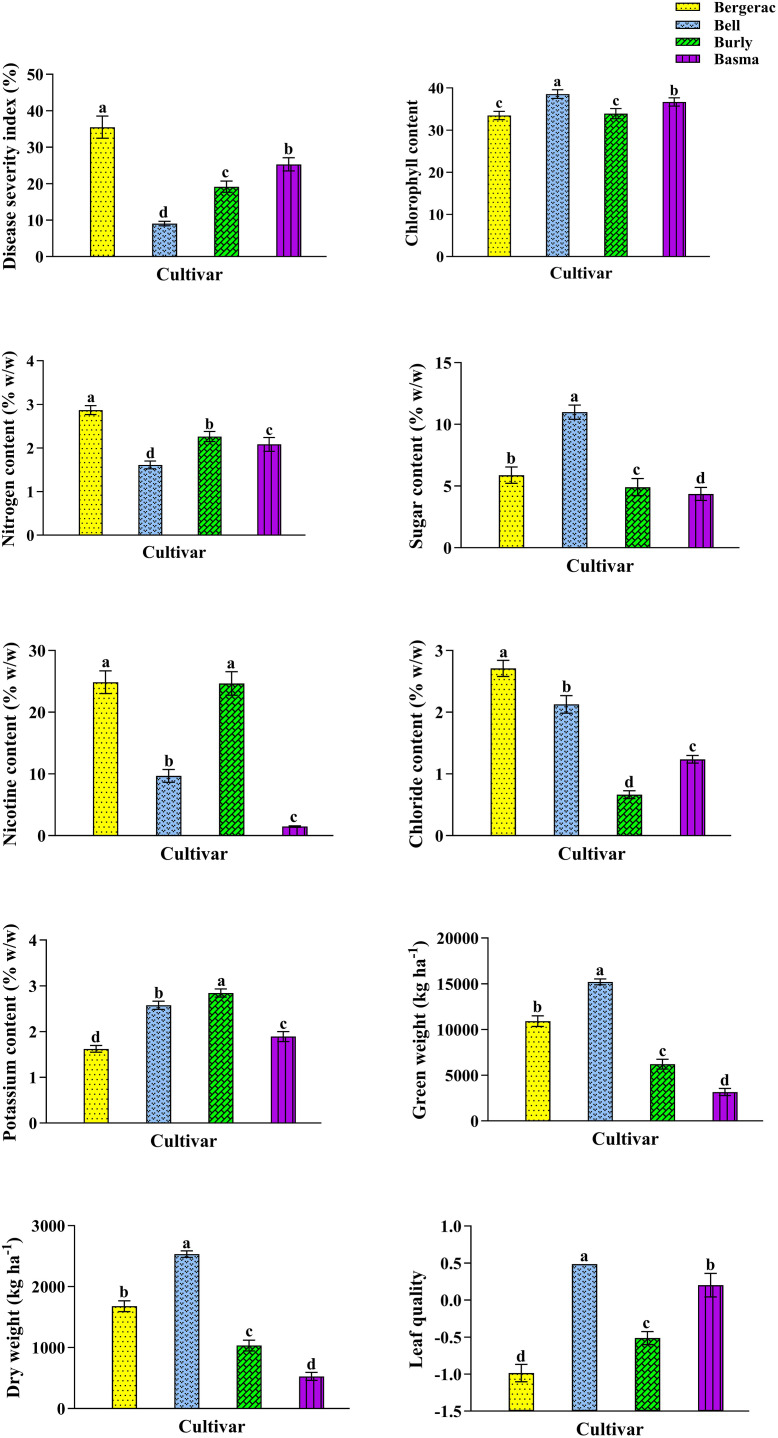
The characteristics of *Nicotiana tabacum* cultivars, including Bergerac, Bell, Burly, and Basma, in 2016 growing season. Average values are given, standard error are represented by vertical lines. Means followed by the same letter are not significantly different (*p ≤ 0.05*).

Subsequently, the lowest BMS in 2016 belonged to Bell, with an average of 9.06% (Fig 1). The highest BMS in 2016 was observed in Bergerac leaves (35.50%) which was 3.92 times that in Bell (Fig 5). Also, the highest Chl content (38.56) was measured in Bell leaves (Fig 5). While, the lowest Chl content in 2016 belonged to Bergerac and Burly leaves with an average of 33.46 and 33.93, respectively (Fig 5). Also, Bergerac leaves had the highest N and Cl with an average of 2.87 and 2.71% (w/w), respectively (Fig 5), which were 78.26 and 310.61% higher than those recorded for Bell and Burly, respectively (Fig 5). Bell and Burly leaves had the lowest content of N and Cl, respectively (Fig 5). Besides, the Bell leaves displayed the highest S (10.99% w/w), GW (15196.62 kg ha^-1^), and DW (2532.77 kg ha^-1^), which were 2.52, 4.80, and 4.79 times S, GW, and DW, respectively, measured for Basma (Fig 5). As shown in Fig 4, the lowest S, GW, and DW belonged to Basma. Also, Bergerac and Burly had the highest Nt (24.76% w/w), which was 16.73 times measured in Basma (Fig 5). The lowest K with an average of 1.62% w/w was measured in Bergerac leaves (Fig 5). The highest K was recorded for Burly leaves, which was 1.75 times measured in Bergerac ones (Fig 5). Additionally, Bell leaves had the highest quality with an average of 0.4864, i.e., 58.73% higher than that in Bergerac (Fig 5). It is important to note that 58.73% is based on the main scale of measurement in our dataset (μ = 1579996.5 Iranian Rials kg^-1^ DW and σ = 452320.3).

### Regression analysis

Initially, BMS, Chl, N, S, Nt, Cl, and K were used as input variables, and tobacco leaf quality was used as the output variable. Then, the output variable was predicted using the developed MLR, SR, OLSR, PCR and PLSR models to find out the foremost regression method for the prediction of tobacco leaf quality responding to BMS, Chl, N, S, Nt, Cl, K. All the mentioned regression methods modeled statistically significant relationships between BMS, Chl, N, S, Nt, Cl, and K (the input variables) and tobacco leaf quality as an output variable (Table 1). Table 1 shows the statistics of MLR, SR, OLSR, PCR, and PLSR models developed for predicting tobacco leaf quality responding to BMS, Chl, N, S, Nt, Cl, and K in Bergerac, Bell, Burly, Basma, and all cultivars. R^2^, a measure of goodness-of-fit, displayed the predictive efficiency of MLR, SR, OLSR, PLSR, and PCR models developed for Bergerac leaf quality in the training subset were 0.74, 0.72, 0.76, 0.73, and 0.76, respectively (Table 1). The prediction accuracies of the regression models for Bell, Burly and Basma leaf quality were: MLR = 42, 49 and 56%, SR = 46, 48 and 54%, OLSR = 50, 51 and 59%, PLSR = 25, 39 and 52%, and PCR = 50, 51 and 59%, respectively (Table 1). MLR, SR, OLSR, PLSR, and PCR models’ accuracies in predicting leaf quality for all cultivars (based on data from all cultivars) in the training subset were 66, 66, 69, 64, and 69%, respectively (Table 1). The developed MLR, SR, OLSR, PLSR, and PCR models explained 78, 78, 82, 77, and 82% of Bergerac leaf quality variability according to R^2^ values for the testing subset (Table 1). Additionally, MLR, SR, OLSR, PLSR, and PCR models accounted for 35, 26, 41, 21, and 41%, respectively, variability in Bell leaf quality (Table 1). Also, R² values for the testing subset indicated that the developed MLR, SR, OLSR, PLSR, and PCR models explained 47, 41, 68, 57, and 68% variability in Burly leaf quality, respectively (Table 1). Besides, MLR, SR, OLSR, PLSR, and PCR accounted for 66, 63, 68, 61, and 68% variance in Basma leaf quality (Table 1). Furthermore, MLR, SR, OLSR, PLSR, and PCR models accounted for 62, 62, 66, 63, and 66%, respectively, variability in leaf quality (based on data from all cultivars, Table 1).

**Table 1:** Statistics on multiple linear regression (MLR), stepwise regression (SR), principal component regression (PCR), ordinary least squares regression (OLSR), partial least squares regression, and multilayer perceptron-genetic algorithm (MLPNN-GA) for modeling leaf quality responding to blue mold severity, chlorophyll, nitrogen, sugar, nicotine, chloride, and potassium content in four tobacco cultivars “Bergerac, Bell, Burly, and Basma”.

Cultivar	Models	Neuron number	Training subsets			Testing subsets			Pr > F
			R^2^	RMSE	MAPE	R^2^	RMSE	MAPE	
**Bergerac**	**MLR**	–	0.74	0.214	14.66	0.78	0.195	13.63	< 0.0001
	**SR**	–	0.72	0.220	14.61	0.78	0.195	13.63	< 0.0001
	**OLSR**	–	0.76	0.214	14.02	0.82	0.193	12.95	< 0.0001
	**PLSR**	–	0.73	0.213	14.51	0.77	0.188	13.59	< 0.0001
	**PCR**	–	0.76	0.214	14.02	0.82	0.193	12.95	< 0.0001
	**MLPNN-GA**	4	**1.00**	**0.000**	**0.00**	**1.00**	**0.00**	**0.00**	< 0.0001
**Bell**	**MLR**	–	0.42	0.140	4.81	0.35	0.148	4.58	< 0.0001
	**SR**	–	0.46	0.136	4.51	0.26	0.155	5.08	< 0.0001
	**OLSR**	–	0.50	0.136	4.52	0.41	0.156	4.61	< 0.0001
	**PLSR**	–	0.25	0.156	5.16	0.21	0.155	4.99	< 0.0001
	**PCR**	–	0.50	0.136	4.52	0.41	0.156	4.61	< 0.0001
	**MLPNN-GA**	6	**1.00**	**0.000**	**0.00**	**1.00**	**0.000**	**0.00**	< 0.0001
**Burly**	**MLR**	–	0.49	0.175	10.99	0.47	0.188	11.61	< 0.0001
	**SR**	–	0.48	0.176	10.94	0.41	0.195	11.83	< 0.0001
	**OLSR**	–	0.51	0.178	10.54	0.68	0.162	9.07	< 0.0001
	**PLSR**	–	0.39	0.186	12.11	0.57	0.162	11.0	< 0.0001
	**PCR**	–	0.51	0.178	10.55	0.68	0.162	9.07	< 0.0001
	**MLPNN-GA**	4	**1.00**	**0.000**	**0.00**	**1.00**	**0.000**	**0.00**	< 0.0001
**Basma**	**MLR**	–	0.56	0.341	13.16	0.66	0.340	16.11	< 0.0001
	**SR**	–	0.54	0.348	13.39	0.63	0.348	16.72	< 0.0001
	**OLSR**	–	0.59	0.343	12.01	0.68	0.366	16.63	< 0.0001
	**PLSR**	–	0.52	0.350	15.19	0.61	0.345	17.11	< 0.0001
	**PCR**	–	0.59	0.343	12.01	0.68	0.366	16.63	< 0.0001
	**MLPNN-GA**	5	**1**	**0.000**	0.00	**0.94**	**0.127**	**7.9**	< 0.0001
**All cultivars**	**MLR**	–	0.66	0.262	14.37	0.62	0.285	14.67	< 0.0001
	**SR**	–	0.66	0.262	14.37	0.62	0.285	14.67	< 0.0001
	**OLSR**	–	0.69	0.256	13.59	0.66	0.277	14.47	< 0.0001
	**PLSR**	–	0.64	0.271	14.48	0.63	0.279	14.37	< 0.0001
	**PCR**	–	0.69	0.256	13.59	0.66	0.277	14.47	< 0.0001
	**MLPNN-GA**	6	**0.97**	0.074	3.79	0.94	0.115	5.52	< 0.0001

R2: coefficient of determination, RMSE: root mean square error, MAPE: mean absolute percentage error

### Multilayer perceptron-genetic algorithm analysis

First, BMS, Chl, N, S, Nt, Cl, and K were used as input variables, and Bergerac, Bell, Burly, and Basma leaf quality were used as output variables. Then, tobacco leaf quality was forecasted using MLPNN-GA models developed based on BMS, Chl, N, S, Nt, Cl, and K as input variables. The prediction made according to the developed MLPNN-GA models closely matched the actual observed data, both for the training and testing subset (Table 1). R^2^, a measure of goodness-of-fit, of the developed MLPNN-GA models for predicting the leaf quality in Bergerac, Bell, Burly, Basma, and all cultivars was 1.00, 1.00, 1.00, 0.94, and 0.94, respectively, in the testing subset (not used in training processes; Table 1), indicating a high accuracy in the prediction process. Additionally, the training (used to train the model initially) and testing (not used during training) subsets displayed balanced statistical values for the developed MLPNN-GA models, which shows that the model is not overfitting and can generalize well to new data.

### Model sensitivity analysis

To list BMS, Chl, N, S, Nt, Cl, and K (input variables) in the developed MLPNN-GA based on their relative importance, VSRs were calculated using all of the data lines (Table 2). Analysis of the developed MLPNN-GA model for Bergerac revealed that its leaf quality was most sensitive to BMS (VSR = 1.000), followed by S (VSR = 0.240), N (VSR = 0.090), K (VSR = 0.080), Chl (VSR = 0.055), Nt (VSR = 0.011), and Cl (VSR = 0.000; Table 2). Accordingly, Bell leaf quality was more sensitive to BMS (VSR = 1.000), followed by the Chl (VSR = 0.990), S (VSR = 0.982), Nt (VSR = 0.027), N (VSR = 0.021), Cl (VSR = 0.000), and K (VSR = 0.000; Table 2). Burly displayed more sensitivity to BMS (VSR = 1.000), followed by the Chl (VSR = 0.537), Nt (VSR = 0.527), N (VSR = 0.457), S (VSR = 0.400), Cl (VSR = 0.002), and K (VSR = 0.000; Table 2). Also, Basma leaf quality showed more sensitivity to BMS (VSR = 1.000), followed by S (VSR = 0.251), Nt (VSR = 0.061), N (VSR = 0.036), Chl (VSR = 0.011), K (VSR = 0.002), and Cl (VSR = 0.000; Table 2). Additionally, leaf quality (based on data from all cultivars) was more sensitive to BMS (VSR = 1.000), followed by the Nt (VSR = 0.290), N (VSR = 0.104), S (VSR = 0.053), Chl (VSR = 0.048), Cl (VSR = 0.025), and K (VSR = 0.000; Table 2).

**Table 2:** Importance (according to the sensitivity analysis) of the input variables, including blue mold severity, chlorophyll, nitrogen, sugar, nicotine, chloride, and potassium content, for the achievement of the maximum leaf quality in four tobacco cultivars, including Bergerac, Bell, Burly, and Basma, using multilayer perceptron neural network-genetics algorithm models (MLPNN-GA).

Variable	Importance value (according to VSR^a^)				
**Bergerac**	**Bell**	**Burly**	**Basma**	**All cultivars**
**Blue mold severity**	1.0000	1.0000	1.0000	1.0000	1.0000
**Chlorophyll content**	0.0546	0.9901	0.5369	0.0110	0.0480
**Nitrogen content**	0.0897	0.0211	0.4568	0.0357	0.1045
**Sugar content**	0.2405	0.9821	0.3992	0.2508	0.0531
**Nicotine content**	0.0110	0.0267	0.5270	0.0612	0.2899
**Chloride content**	0.0000	0.0000	0.0015	0.0000	0.02474
**Potassium content**	0.0803	0.0000	0.0000	0.0018	0.0000

^a^Relative indication of the ratio between the variable sensitivity error and the error of the model when all variables are available.

### Comparison of MLPNN-GA and regression models

MLPNN-GA models displayed higher prediction accuracy compared with regression models according to R^2^ for MLPNN-GA vs. regression models were: Bergerac; 1.00 vs. 0.82, Bell = 1.00 vs. 0.41, Burly = 1.00 vs. 0.68, Basma = 0.94 vs. 0.68, and all cultivars = 0.94 vs. 0.66 (Table 1). It is worth noting that the R-squared of the best regression models was compared to MLPNN-GA.

## Discussion

Precise analysis of the effects of the factors determining tobacco leaf quality and finding their relative importance on leaf quality would pave the way for improving the farmer’s income in low- and middle-income countries. Developed MLPNN models predicting tobacco leaf prices are valuable for farmers in different ways: farmers’ income increase: By accurately predicting the value of their crops before selling, farmers can negotiate better prices, avoid underselling, and identify high-value leaves. Cultivation practice optimization: Understanding which chemical compounds increase market value enables farmers to adjust their fertilization, irrigation, and harvesting methods to improve crop quality. Market transparency: An AI-driven pricing tool introduces fairness and clarity in pricing, helping to eliminate middlemen who exploit farmers by undervaluing their products. Access to premium and export markets: With reliable quality data, farmers can confidently enter higher-end markets and meet the standards required by major tobacco companies and international buyers. Support for agricultural policy: The model provides valuable data that can guide governments and agricultural organizations in offering targeted support, subsidies, and training programs to enhance agricultural productivity. Indeed, policymakers can use this information to identify high-value crops, prioritize regions for support, and distribute subsidies or training programs more effectively. These models lead to brighter, fairer, and more sustainable agricultural development, ultimately improving the livelihoods of farmers and the efficiency of national agricultural strategies.

This report presents the first mathematical model development for tobacco leaf quality prediction based on BMS, Chl, N, S, Nt, Cl, K. A variety of regression models, including MLR, SR, OLSR, PLSR, and PCR, and MLPNN-GA modeling were applied to study the relationships among the input variables “BMS, Chl, N, S, Nt, Cl, K” and the output variable “leaf quality” and the prediction probability of leaf quality using the mentioned input variables. No mathematical predictions have been reported for modeling tobacco leaf quality responding to BMS, Chl, N, S, Nt, Cl, K. The previous research [25–27,30,35,57] has shown that MLPNN-GA outperformed regression models in terms of prediction accuracy. The best regression models (PCR and OLSR) were able to explain 82, 41, 68, 68, and 66% of the leaf quality variability in Bergerac, Bell, Burly, Basma, and all cultivars, respectively, according to the R-squared of the testing subset. Our finding indicated that the MLPNN-GA models were able to account for 100, 100, 100, 94, and 94% of the variation observed in leaf quality of Bergerac, Bell, Burly, Basma, and all cultivars, respectively (Table 1), in the testing subset, which was not included during the training process. The lack of overlearning during training and the strong generalizability of the developed MLPNN-GA models for unknown data were validated by the proximity of the errors of the training and testing subsets and the modest number of hidden neurons (Table 1). The training and testing subsets’ statistical parameters, R^2^, RMSE, and MAPE (Table 1), showed that the tansig activation function was a good one for modeling throughout the study.

Despite the previous research on the effects of blue mold severity, sugar, chloride, nicotine, potassium, and total nitrogen contents of tobacco leaves on their quality, the question still stands: which input factors are most important for tobacco leaf quality? The most significant factor influencing tobacco leaf quality, as previously indicated by sensitivity analysis, was BMS (Table 2). Tobacco leaf quality is a complex phenomenon affected by genotype (cultivar), environment (year), and their interaction that calls for accurate modeling techniques. In many different fields, MLPNN-GA has proven to be an effective tool for solving problems with incredibly complex and unknown solutions [27,35,57]. Multilayer Perceptrons (MLPs) excel in handling complex, non-linear relationships in data, offering flexibility and scalability for large datasets. They are well-suited for problems where learning intricate patterns is crucial. MLPs utilize specific learning algorithms to optimize themselves as they receive updated inputs, allowing for continuous improvement. In contrast, Support Vector Machines (SVMs) are more efficient for smaller datasets, high-dimensional data, and situations where robustness to outliers and interpretability are paramount [58]. MLPs can often generalize better to unseen data compared to Random Forests, especially when the training data has complex patterns. Additionally, MLPs can often generalize better to unseen data compared to Random Forests, especially when the training data has complex patterns [59,60]. ANNs have been receiving remarkably growing attention due to their power to model complex and nonlinear relationships in various fields, their capability to predict relationships in unseen data, and the certainty that they do not require any assumptions about statistical data distributions [27,57,61]. The high prediction efficiency of the testing subset (Table 1) showed that the developed MLPNN-GA models precisely predicted the leaf quality of Bergerac, Bell, Burly, and Basma.

## Conclusion

In this study, the effects of two growing seasons and four cultivars were evaluated on blue mold severity, chlorophyll, nitrogen, sugar, nicotine, chloride, and potassium content, as well as green weight, dry weight, and quality of *N. tabacum* leaves. Also, the leaf quality of Bergerac, Bell, Burly, and Basma was modeled using mathematical methods for the first time. The close match between the predicted and actual data, for both training and testing subsets, validated the superior efficiency of the developed MLNNP-GA models for predicting Bergerac, Bell, Burly, and Basma leaf quality responding to BMS, Chl, N, S, Nt, Cl, K. This research presents MLPNN-GA as a practical mathematical tool for predicting complex phenomena, including the tobacco leaf quality responding to its chemical components. To improve future predictions, it is recommended to expand the dataset across different regions and seasons, include more agronomic and environmental variables, and explore ensemble deep learning methods. Such enhancements could increase model generalizability and support more accurate, data-driven decisions in the tobacco industry.

## Supporting information

S1 File Four cultivars (Bergerac, Bell, Burly, and Basma) on the field condition.
(DOCX)
