# Supplementary material for: Multilayer perceptron neural network-genetic algorithm for modeling Nicotiana tabacum leaf quality
Source: PLoS One. 2025 Oct 7;20(10):e0330370. doi: 10.1371/journal.pone.0330370 (PMC12503340; doi:10.1371/journal.pone.0330370)
Supplement: S1 File — Four cultivars (Bergerac, Bell, Burly, and Basma) on the field condition. (DOCX) [file pone.0330370.s001.docx]

**Multilayer perceptron neural network-genetic algorithm for modeling *Nicotiana tabacum* leaf quality**

**Mohammad Reza Najafi^1^, Mohammad Ali Aghajani^2^, Naser Safaie^3*^, Siamak Rahmanpour^4^**

1. Department of Plant Protection, College of Agriculture Sciences and Food Industries, Science and Research Branch, Islamic Azad University, Tehran, Iran

2. Plant Protection Research Department, Golestan Agricultural and Natural Resources Research Center, AREEO, Gorgan, Iran

3. Department of Plant Pathology, Faculty of Agriculture, Tarbiat Modares University, P.O. Box 14115‑336, Tehran, Iran

4. Department of Oil Seed Research, Seedling and Seed Research Institute, Karaj, Iran

*Correspondence: Naser Safaie; nsafaie@modares.ac.ir


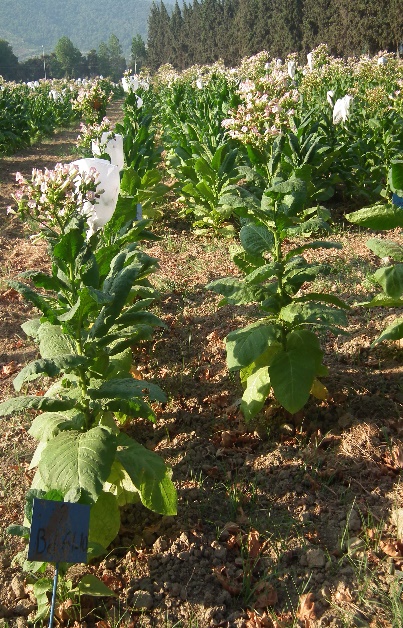

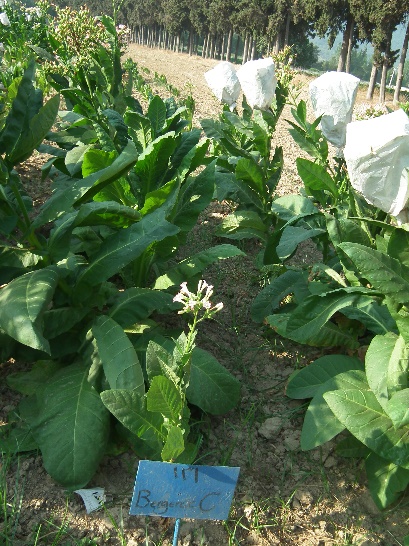

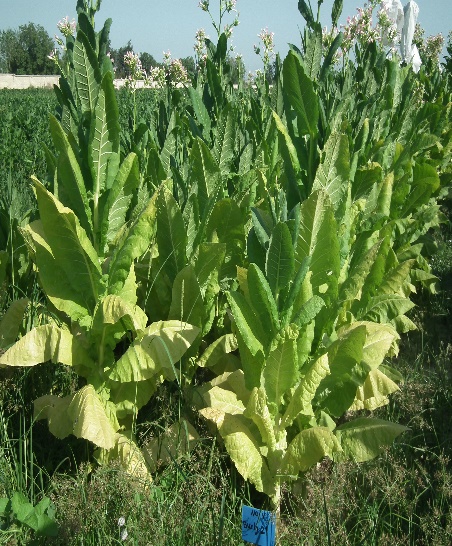

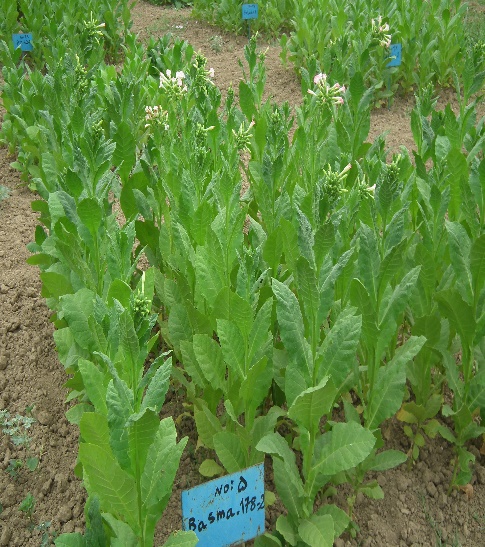


**Bergerac**

**Bell**

**Burly**

Basma

**Figure S1.** Four of *Nicotiana tabacum* cultivars cultivars (Bergerac, Bell, Burly, and Basma) on the field condition

| **Table S1.** Combined analysis of variance of the characteristics of *Nicotiana tabacum* cultivars, including Bergerac, Bell, Burly, and Basma, in two growing seasons (2015 and 2016) | | | | | | | | | | | | |
| --- | --- | --- | --- | --- | --- | --- | --- | --- | --- | --- | --- | --- |
| Source of variation | Degree of freedom | Disease severity | Chlorophyll Content | Nitrogen content | Sugar content | Nicotine content | Chloride content | Potassium content | Green weight | Dry weight | Quality |  |
| Year | 1 | 7534.2^**^ | 4832.3^**^ | 0.378^ns^ | 8.62^ns^ | 282.0^*^ | 0.111^ns^ | 9.831^**^ | 1174833893^**^ | 60292615.8^**^ | 16.458^**^ |  |
| Block (year) | 98 | 471.0^**^ | 199.9^**^ | 2.566^**^ | 53.45^**^ | 260.6^**^ | 0.885^**^ | 0.586^ns^ | 48814425^**^ | 1445440.9^**^ | 1.318^**^ |  |
| Cultivar | 3 | 8927.5^**^ | 656.3^**^ | 18.660^**^ | 1360.48^**^ | 13338.2^**^ | 67.516^**^ | 28.505^**^ | 2403495553^**^ | 78403527.5^**^ | 46.926^**^ |  |
| Cultivar × Year | 3 | 273.8^**^ | 42.9^**^ | 3.841^**^ | 268.82^**^ | 635.5^**^ | 2.763^**^ | 1.357^*^ | 150457470^**^ | 1748072.7^**^ | 1.717^**^ |  |
| Error | 294 | 48.1 | 4.1 | 0.106^**^ | 3.15 | 37.0^**^ | 0.429 | 0.505 | 2422182 | 59656.3 | 0.369 |  |
| Coefficient of Variation (CV) |  | 38.7 | 5.2 | 14.5 | 26.5 | 38.0 | 39.3 | 29.7 | 14.7 | 13.3 | 17.4 |  |
| *, ** and ns indicate significant difference *p*<0.05, significant difference *p*<0.01 and non-significant, respectively. | | | | | | | | | | | | |

| **Table S2.** Analysis of variance of the characteristics of *Nicotiana tabacum* cultivars, including Bergerac, Bell, Burly, and Basma, in 2015 growing season | | | | | | | | | | | |
| --- | --- | --- | --- | --- | --- | --- | --- | --- | --- | --- | --- |
| Source of variation | Degree of freedom | Disease severity | Chlorophyll Content | Nitrogen content | Sugar content | Nicotine content | Chloride content | Potassium content | Green weight | Dry weight | Price |
| Block | 49 | 365.8** | 192.6** | 2.621^**^ | 36.2** | 230.9^**^ | 1.036^**^ | 0.594^ns^ | 58681715^**^ | 1862132.2^**^ | 1.42^**^ |
| Cultivar | 3 | 3061.4** | 412.4** | 8.995^**^ | 1168.9^**^ | 7284.0^**^ | 28.758^**^ | 13.520^**^ | 1156681459^**^ | 42701974.3^**^ | 26.18^**^ |
| Error | 147 | 34.7 | 2.8 | 0.091 | 4.5 | 35.1 | 0.358 | 0.648 | 3110733 | 76847.4 | 0.37 |
|  | - | 43.4 | 4.0 | 13.3 | 31.0 | 35.1 | 36.3 | 31.6 | 14.3 | 12.5 | 16.4 |
| *, ** and ns indicate significant difference *p*<0.05, significant difference *p*<0.01 and non-significant, respectively. | | | | | | | | | | | |

| **Table S3.** Analysis of variance of the characteristics of *Nicotiana tabacum* cultivars, including Bergerac, Bell, Burly, and Basma, in 2016 growing season | | | | | | | | | | | |
| --- | --- | --- | --- | --- | --- | --- | --- | --- | --- | --- | --- |
| Source of variation | Degree of freedom | Disease severity | Chlorophyll Content | Nitrogen content | Sugar content | Nicotine content | Chloride content | Potassium content | Green weight | Dry weight | Price |
| Block | 49 | 576.1^**^ | 207.3^**^ | 2.512^**^ | 70.7^**^ | 290.2^**^ | 0.734^*^ | 0.578^*^ | 38947135^**^ | 1028749.5^**^ | 1.21^**^ |
| Cultivar | 3 | 6139.8^**^ | 286.7^**^ | 13.507^**^ | 460.4^**^ | 6689.8^**^ | 41.522^**^ | 16.343^**^ | 1397271564^**^ | 37449625.9^**^ | 22.46^**^ |
| Error | 147 | 61.6 | 5.3 | 0.121 | 1.8 | 38.9 | 0.500 | 0.362 | 1733631 | 42465.1 | 0.37 |
|  |  | 35.3 | 6.4 | 15.8 | 20.6 | 41.1 | 42.0 | 26.9 | 14.8 | 14.3 | 18.5 |
| *, ** and ns indicate significant difference *p*<0.05, significant difference *p*<0.01 and non-significant, respectively. | | | | | | | | | | | |
